# Chromatin Remodeling Complex SWR1 Regulates Root Development by Affecting the Accumulation of Reactive Oxygen Species (ROS)

**DOI:** 10.3390/plants12040940

**Published:** 2023-02-19

**Authors:** Youmei Huang, Xinpeng Xi, Mengnan Chai, Suzhuo Ma, Han Su, Kaichuang Liu, Fengjiao Wang, Wenhui Zhu, Yanhui Liu, Yuan Qin, Hanyang Cai

**Affiliations:** 1 Fujian Provincial Key Laboratory of Haixia Applied Plant Systems Biology, State Key Laboratory of Ecological Pest Control for Fujian and Taiwan Crops, College of Life Sciences, Fujian Agriculture and Forestry University, Fuzhou 350002, China; 2 College of Life Science, Longyan University, Longyan 364012, China; 3 State Key Laboratory for Conservation and Utilization of Subtropical Agro-Bioresources, Guangxi Key Lab of Sugarcane Biology, College of Agriculture, Guangxi University, Nanning 530004, China

**Keywords:** ROS, root, SWR1, *Arabidopsis*, H2A.Z, oxidoreductase activity-related genes

## Abstract

Reactive oxygen species (ROS), a type of oxygen monoelectronic reduction product, play integral roles in root growth and development. The epigenetic mechanism plays a critical role in gene transcription and expression; however, its regulation of ROS metabolism in root development is still limited. We found that the chromatin remodeling complex SWR1 regulates root length and lateral root formation in *Arabidopsis*. Our transcriptome results and gene ontology (GO) enrichment analysis showed that the oxidoreductase activity-related genes significantly changed in mutants for the *Arabidopsis* SWR1 complex components, such as *arp6* and *pie1*, and histone variant H2A.Z triple mutant *hta8 hta9 hta11.* The three encoding genes in *Arabidopsis* are the three H2A.Z variants *hta8*, *hta9*, and *hta11*. Histochemical assays revealed that the SWR1 complex affects ROS accumulation in roots. Furthermore, chromatin immunoprecipitation quantitative real-time PCR (ChIP-qPCR) analysis showed that the reduced H2A.Z deposition in oxidoreductase activity-related genes caused ROS to accumulate in *arp6*, *pie1*, and *hta8 hta9 hta11*. H2A.Z deposition-deficient mutants decreased after the trimethylation of lysine 4 on histone H3 (H3K4me3) modifications and RNA polymerase II (Pol II) enrichment, and increased after the trimethylation of lysine 27 on histone H3 (H3K27me3) modifications, which may account for the expression change in oxidoreductase activity-related genes. In summary, our results revealed that the chromatin complex SWR1 regulates ROS accumulation in root development, highlighting the critical role of epigenetic mechanisms.

## 1. Introduction

Roots are important plant organs, playing an essential role in plant growth and development. Most plant roots grow in soil, forming the plant’s underground world and providing physical support and fixation for the plant. In addition, roots can absorb water and nutrients from the soil and transport them to the aerial part through their vascular tissue [1,2]. In higher plants, plant root growth depends on maintaining the balance between the proliferation and differentiation of cells in root [3,4]. 

Roots are divided into three regions along the longitudinal axis: the meristematic, elongation, and maturation zones [5]. In the meristematic zone, the cells are small, closely arranged, and have a high rate of cell division. In the elongation zone, most cells stop dividing and elongating [6]. The transition from cellular proliferation to elongation marks the initial stage of differentiation, producing a boundary called the transition zone (TZ) [7]. The cells in the mature zone have completed elongation and differentiation, and the epidermal cells exhibit root hairs [1,8,9]. Lateral roots (LRs) are newly formed organs in the maturation zone, which originate from a subset of pericyclic cells called pericyclic initiating cells in the vascular tissues of the maturation root region of the plant [10,11]. Root growth and development are affected by many factors, including the redox environment and epigenetic regulation [3,12,13].

Reactive oxygen species (ROS) are oxygen-containing molecules or ions with active chemical properties, such as the superoxide radical (O^2−^), hydroxyl radical (OH^−^), and hydrogen peroxide (H_2_O_2_). They are continuously produced in normal aerobic metabolic processes, including photosynthesis and respiration [14,15,16,17]. Available evidence reveals that ROS are important and versatile signaling molecules involved in plant growth and development, including programmed cell death, the hormone signaling pathway, and biotic and abiotic stress responses [18,19,20,21]. Several studies have revealed that ROS maintain the balance between cell proliferation and differentiation in the root [1,22]. For example, root meristem growth factor 1 (RGF1) is an essential peptide hormone that controls root meristem size through ROS signaling [23,24]. MYB30 is one of the key transcription regulators of ROS signal transduction that regulates the meristem zone and root cell elongation [25].

Epigenetic regulation can change the structure of chromatin through different modification methods, such as DNA methylation, histone modification, and chromatin remodeling, changing the gene expression level and regulating the specialization and fate of cells without altering the gene sequence [26,27,28]. Previous studies have shown that the nucleosome is a dynamic structure involving different nucleosome characteristics that change gene expression and function [29,30]. The chromatin remodeling complex SWR1 is essential for plant vegetative and reproductive growth, using ATP hydrolysis energy to catalyze the replacement of histone H2A-H2B dimers with H2A.Z-H2B dimers, affecting the gene’s transcription factor binding [31,32,33,34]. H2A.Z is a highly conserved histone variant [35]. In *Arabidopsis*, three genes encode H2A.Z: *HISTONE H2A 8*(*HTA8*), *HTA9*, and *HTA11* [30]. To date, some subunits of *Arabidopsis* SWR1 complex have been reported, including PHOTOPERIOD-INDEPENDENT EARLY FLOWERING 1 (PIE1), ACTIN-RELATED PROTEIN 6 (ARP6), SERRATED LEAVES AND EARLY FLOWERING (SEF), SWR1 COMPLEX SUBUNIT 2 (SWC2), SWC4, and METHYL-CpG-BINDING DOMAIN9 (MBD9) [30,36,37,38]. Increasing evidence suggests that SWR1 is associated with biotic and abiotic stress responses [39,40]. SWR1 also plays an important role in flowering time, flower architecture, and hypocotyl elongation [31,41,42]. Furthermore, the role of SWR1 in megasporocyte cell fate is well-established [43]. However, the function of SWR1 in regulating root growth and development is still unclear.

In this study, we showed that H2A.Z-deficient mutant plants exhibit root growth arrest and fewer LRs than those in wild-type (WT) plants. Transcriptome data and histochemical assays showed that ROS accumulation increased in the roots of H2A.Z deposition-deficient mutants. We also found that oxidoreductase activity-related genes’ reduced expression was associated with decreased H2A.Z deposition, H3K4me3 histone modification, RNA Pol II enrichment, and increased H3K27me3 histone modification in H2A.Z deposition-deficient mutants. Our study revealed that the chromatin SWR1 complex plays an important role in regulating gene expression in root growth and development.

## 2. Results

### 2.1. SWR1 Complex Is Involved in Root Length and LR Formation

**Figure 1:**
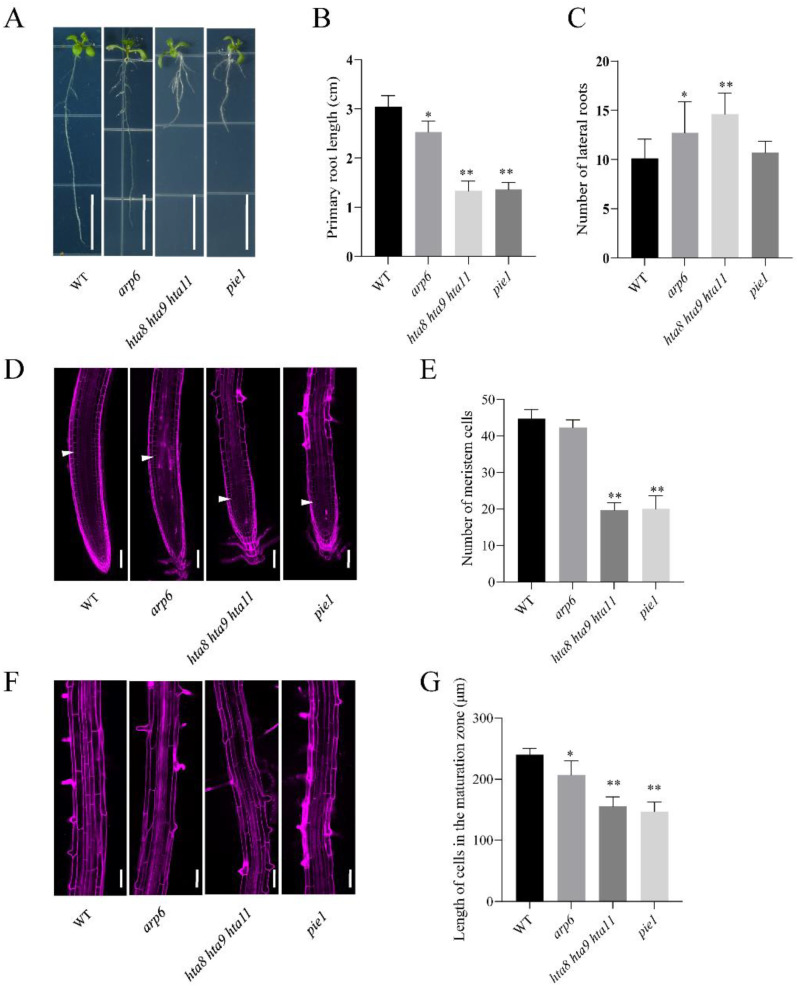
*Arabidopsis* H2A.Z deposition-deficient mutants show altered root length and lateral root formation: (**A**) Phenotypes of WT and H2A.Z deposition-deficient mutants *arp6*, *hta8 hta9 hta11*, and *pie1* at 10 days after germination. Bar = 1 cm. (**B**) The primary root length of WT, *arp6*, *hta8 hta9 hta11*, and *pie1* at 10 days after germination. Bars show standard deviations of at least 10 seedlings. Asterisks indicate significant differences between the WT and each mutant evaluated with Student’s *t* test (* *p* < 0.05, ** *p* < 0.01). Outlier values are represented by dots. (**C**) The lateral root number of WT, *arp6*, *hta8 hta9 hta11*, and *pie1* at 10 days after germination. Bars show standard deviations of at least 10 seedlings. Asterisks indicate significant differences between the WT and each mutant evaluated with Student’s *t* test (* *p* < 0.05, ** *p* < 0.01). (**D**) Confocal images of root tips of WT, *arp6*, *hta8 hta9 hta11*, and *pie1* with propidium iodide (Pi) staining. White arrows indicate the position above which is the first elongated cell. Bar = 100 µm. (**E**) Number of meristem cells. The cortex cell number between the quiescent center and the first elongated cell was countered. At least 10 plants were evaluated per biological repeat. The error bars represent the standard errors of triplicate experiments. ** *p* < 0.01. (**F**) Confocal images of root maturation zone of WT, *arp6*, *hta8 hta9 hta11*, and *pie1* with Pi staining. Bar = 100 µm. (**G**). Length of cells in the maturation zone. At least 10 plants were evaluated per biological repeat. The error bars represent the standard errors of triplicate experiments. * *p* < 0.05, ** *p* < 0.01.

The chromatin remodeling complex SWR1 has been shown to regulate female gametophyte development, inflorescence architecture, and hypocotyl elongation [32,42,44,45]. However, the role of SWR1 in root growth and development is still unclear. To explore the functions of SWR1 in root growth and development, WT and the H2A.Z deposition-deficient mutants *arp6*, *pie1*, and *hta8 hta9 hta11* were grown vertically in MS medium and analyzed in detail. Our results showed that the primary root length of *arp6*, *hta8 hta9 hta11*, and *pie1* were shorter than in WT (Figure 1A,B). We also noticed that *arp6*, *hta8 hta9 hta11*, and *pie1* mutations had more LR than WT (Figure 1A,C). Root elongation rates are accompanied by increased cell production and expansion [6]. We counted the cell number and length using propidium iodide (PI) staining to investigate the causes of root length change. Our results showed that *arp6*, *hta8 hta9 hta11*, and *pie1* had fewer meristem cells than WT (Figure 1D,E). Compared with WT, *arp6* and especially *hta8 hta9 hta11* and *pie1* had shorter cell lengths in the maturation zone (Figure 1F,G). These results suggest that the key components of SWR1 and the H2A.Z histone variant play important roles in plant root growth and development.

### 2.2. Transcriptome Data Reveal the Potential Role of SWR1 Complex in Root Growth and Development

**Figure 2:**
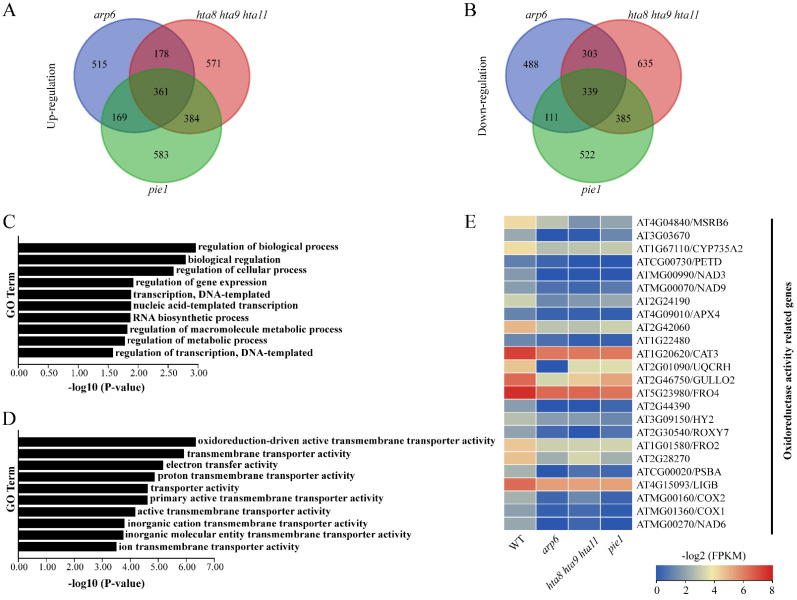
Transcriptomic analysis of WT, *arp6*, *hta8 hta9 hta11*, and *pie1* roots: (**A**,**B**) Venn diagrams show the number of upregulated genes (**A**) and downregulated genes (**B**) in the roots of *arp6*, *hta8 hta9 hta11*, and *pie1* compared to WT. (**C**,**D**) GO enrichment analysis of upregulated genes (**C**) and downregulated genes (**D**). (**E**) Expression pattern of oxidoreductase activity-related genes in WT, *arp6*, *hta8 hta9 hta11*, and *pie1* roots.

We analyzed the transcriptome data of WT, *arp6*, *hta8 hta9 hta11*, and *pie1* root by RNA-seq to study the potential mechanism of SWR1 complex in root growth and development. We constructed 12 cDNA libraries with three biological replicates. The total mapping rate of the sample ranged from 95.85 to 97.23%. The unique mapping rate ranged from 81.95 to 89.72%, and the multiple mapping rate ranged from 7.44 to 14.07%. These libraries yielded 1.01 to 1.91 Gb raw reads, and the sequencing depth ranged from 8.56 to 16.21× (Table S1). We also found that 361 genes were upregulated and 339 genes were downregulated in *arp6*, *hta8 hta9 hta11*, and *pie1* compared with the corresponding gene in WT (Figure 2A,B and Table S2). Among the 361 upregulated genes, there were 6 protein kinases and 44 transcription factors (Figure S1A,B). The 339 downregulated genes included 13 protein kinases and 26 transcription factors (Figure S1C,D). According to the GO enrichment analysis, 361 upregulated genes were enriched in regulation of biological process, biological regulation and regulation of cellular process (Figure 2C). The 339 downregulated genes were mainly concentrated in oxidoreduction-driven active transmembrane and transmembrane transporter activity and electron transfer activity (Figure 2D). Considering the enrichment of oxidoreductase activity-related genes among downregulated genes, we generated an expression heatmap of these genes in *arp6*, *hta8 hta9 hta11*, and *pie1* to compare to WT. The expression of the oxidoreductase activity-related genes was decreased in *arp6*, *hta8 hta9 hta11*, and *pie1* compared with WT (Figure 2E).

**Figure 3:**
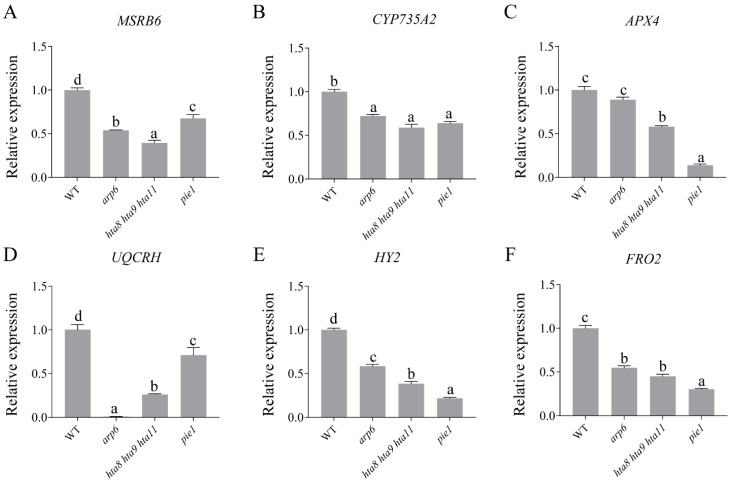
Quantitative real-time PCR expression analysis of six oxidoreductase activity-related genes in the roots of WT, *arp6*, *hta8 hta9 hta11*, and *pie1*: (**A**–**F**) The expression levels of the six selected genes were obtained by qRT-PCR. The error bars indicate ± SD (*n* = 3 replicates). Different letters above the columns indicate significant differences at *p* < 0.05, as determined by one-way ANOVA.

To verify the accuracy of the RNA-seq data, we performed quantitative reverse transcription PCR (qRT-PCR) to compare the expression level of six selected genes in *arp6*, *hta8 hta9 hta11*, and *pie1* to WT, including *METHIONINE SULFOXIDE REDUCTASE B6* (*MSRB6)*, *CYTOCHROME P450* (*CYP735A2*), *ASCORBATE PEROXIDASE 4* (*APX4*), *UBIQUINOL-CYTOCHROME C REDUCTASE HINGE PROTEIN* (*UQCRH*), *ELONGATED HYPOCOTYL 2/GENOMES UNCOUPLED 3* (*HY2/GUN3*), and *FERRIC CHELATE REDUCTASE DEFECTIVE 1/FERRIC REDUCTION OXIDASE 2* (*FRD1/ FRO2)*. Previous studies found that *MSRB6* [46], *CYP735A2* [47,48], *APX4* [49], *UQCRH* [50], *HY2*, and *FRO2* are involved in the oxidation–reduction process [51,52,53]. Gene mutations affect the electron transfer chain or redox reaction in plants, leading to the accumulation of ROS. We found that these six genes’ expression levels were inhibited in *arp6*, *hta8 hta9 hta11*, and *pie1* compared with those in WT (Figure 3A–F), which was consistent with the RNA-seq results. These results indicated the reliability of the RNA-seq data.

### 2.3. SWR1 Complex Affects the Accumulation of ROS in Roots 

**Figure 4:**
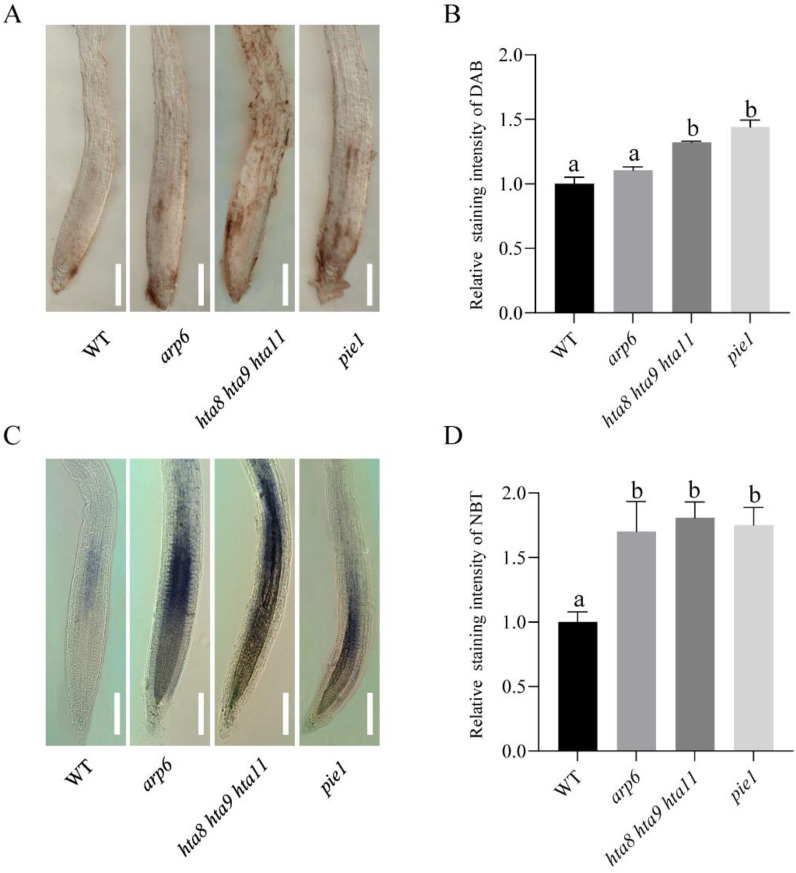
ROS distribution in the roots of 10-day WT, *arp6*, *hta8 hta9 hta11*, and *pie1*: (**A**) DAB staining for H_2_O_2_ in primary root, bar = 100 µm. (**B**) Quantification of DAB staining intensity. The error bars indicate ± SD (*n* > 10 seedlings). Asterisks indicate significant differences for comparisons based on Student’s *t* test (* *p* < 0.05). (**C**) NBT staining for O_2_^−^ in primary root, bar = 100 µm. (**D**) Quantification of NBT staining intensity. The error bars indicate ± SD (*n* > 10 seedlings). Different letters above the columns indicate significant differences at *p* < 0.05, as determined by one-way ANOVA.

To confirm whether the SWR1 complex affects ROS accumulation in roots, hydrogen peroxide (H_2_O_2_), and superoxide (O_2_^−^) were monitored by DAB staining and NBT staining, respectively. Compared with WT, *arp6*, *hta8 hta9 hta11*, and *pie1* seedlings accumulated more H_2_O_2_ in the meristem region (Figure 4A,B). In the meristem and elongation zones, *arp6*, *hta8 hta9 hta11*, and *pie1* accumulated more O_2_^−^ than WT (Figure 4C,D). These results suggest that the key SWR1 and H2A.Z histone variant components are involved in ROS accumulation in roots.

### 2.4. H2A.Z and Pol II Deposition at Oxidoreductase Activity-Related Genes Was Altered in arp6, hta8 hta9 hta11, and pie1

**Figure 5:**
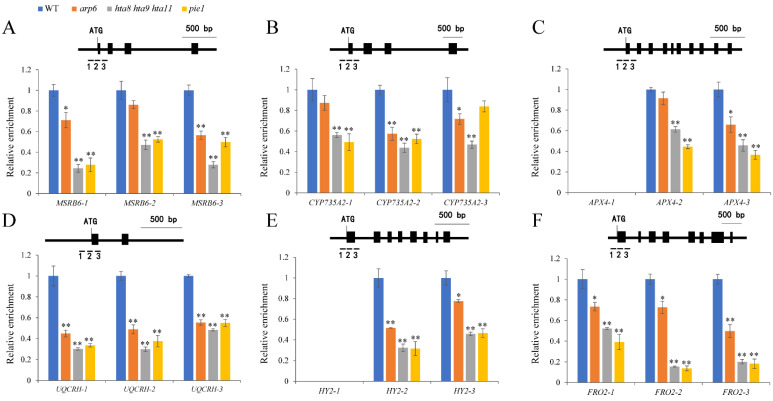
H2A.Z deposition in oxidoreductase activity-related genes was altered in the roots of *arp6*, *hta8 hta9 hta11*, and *pie1*: (**A**–**F**) ChIP-qPCR analysis of H2A.Z deposition at *MSRB6*, *CYP735A2*, *APX4*, *UQCRH*, *HY2*, and *FRO2* genes in WT roots, *arp6*, *hta8 hta9 hta11*, and *pie1*. The exons are indicated by black boxes and the promoter by the region before the ATG indicator. The transcription start site (TSS) is represented by a black indicator line. PCR primer sets are represented by black bars below the diagram. Error bars indicate ± SD (*n* = 3 replicates). Asterisks above the columns indicate statistically significant differences (* *p* < 0.05, ** *p* < 0.01) determined by Student’s *t* test.

In plants, the ATP-dependent SWR1 chromatin remodeling complex regulates gene transcription and expression by modifying the chromatin structure of its target genes by exchanging H2A with H2A.Z [34]. The SWR1 subunits’ mutations lead to a global reduction in H2A.Z abundance on the genome [32]. Our preceding results showed that oxidoreductase activity-related gene expression was significantly decreased in *arp6*, *hta8 hta9 hta11*, and *pie1* seedlings compared with WT. To further investigate whether SWR1 regulates oxidoreductase activity-related genes expression by affecting H2A.Z deposition at their gene loci, we performed a ChIP assay using H2A.Z antibodies to detect the H2A.Z deposition level in the −1 to +1 nucleosome position near the transcription start sites (TSSs) of *MSRB6*, *CYP735A2*, *APX4*, *UQCRH*, *HY2*, and *FRO2*. Our results showed that the enrichment of H2A.Z in the TSS region and ±1 nucleosome of *MSRB6*, *CYP735A2*, *APX4*, *UQCRH*, *HY2*, and *FRO2* was significantly decreased in *arp6*, and especially in *hta8 hta9 hta11*, and *pie1* (Figure 5A–F). 

**Figure 6:**
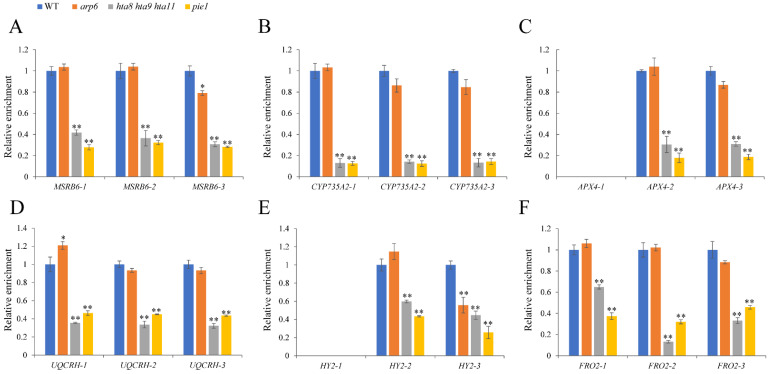
The Pol II level in oxidoreductase activity-related genes was altered in the roots of *arp6*, *hta8 hta9 hta11*, and *pie1*: (**A**–**F**) ChIP-qPCR analysis of Pol II enrichment in *MSRB6*, *CYP735A2*, *APX4*, *UQCRH*, *HY2*, and *FRO2* genes in the roots of WT, *arp6*, *hta8 hta9 hta11*, and *pie1*. Primer set numbers are the same as those listed in Figure 5. Error bars indicate ± SD (*n* = 3 replicates). Asterisks above the columns indicate statistically significant differences (* *p* < 0.05, ** *p* < 0.01) determined by Student’s *t* test.

RNA polymerase II (Pol II) enrichment relates to gene transcription, and several studies have reported that H2A.Z deposition affects Pol II enrichment [54]. Therefore, we performed ChIP-qPCR using the Pol II antibody and *arp6*, *hta8 hta9 hta11*, and *pie1* roots. The enrichment of Pol II was not altered in *MSRB6*, *CYP735A2*, *APX4*, *UQCRH*, *HY2*, and *FRO2* in *arp6* compared with WT. However, the Pol II enrichment in these gene regions was significantly reduced in *hta8 hta9 hta11*, and *pie1* (Figure 6A–F). These results indicated that the enrichment of H2A.Z and Pol II in *MSRB6*, *CYP735A2*, *APX4*, *UQCRH*, *HY2*, and *FRO2* was affected by the key component of SWR1. 

### 2.5. The H3K4me3 and H3K27me3 Levels of Oxidoreductase Activity-Related Genes Were Altered in arp6, hta8 hta9 hta11, and pie1

**Figure 7:**
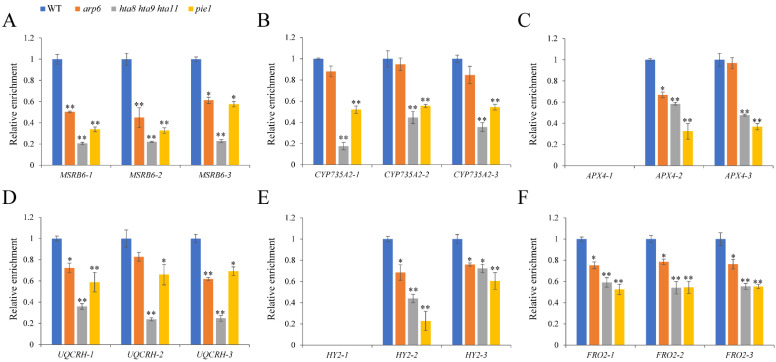
H3K4me3 enrichment in oxidoreductase activity-related genes was altered in *arp6*, *hta8 hta9 hta11*, and *pie1* roots: (**A**–**F**) ChIP-qPCR analysis of the H3K4me3 enrichment in *MSRB6*, *CYP735A2*, *APX4*, *UQCRH*, *HY2*, and *FRO2* genes in WT, *arp6*, *hta8 hta9 hta11*, and *pie1* roots. Primer set numbers are the same as those listed in Figure 5. Error bars indicate ± SD (*n* = 3 replicates). Asterisks above the columns indicate statistically significant differences (* *p* < 0.05, ** *p* < 0.01) determined by Student’s *t* test.

Histone modification plays an important role in regulating gene expression, which promotes or inhibits H2A.Z deposition at different gene loci [55]. To investigate whether the SWR1 complex affects H3K4me3 modification at oxidoreductase activity-related genes, we performed ChIP-qPCR to compare the H3K4me3 enrichment of WT, *arp6*, *hta8 hta9 hta11*, and *pie1*. Compared with WT, H3K4me3 enrichment in *MSRB6*, *CYP735A2*, *APX4*, *UQCRH*, *HY2*, and *FRO2* was reduced in *arp6*, and significantly reduced in *hta8 hta9 hta11*, and *pie1* (Figure 7A–F). 

**Figure 8:**
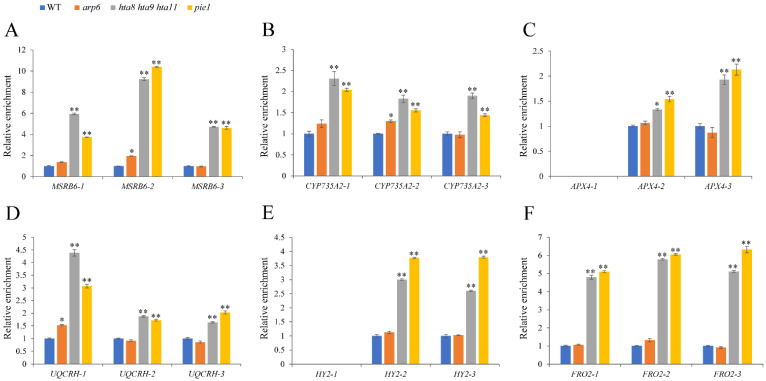
H3K27me3 enrichment in oxidoreductase activity-related genes was altered in *arp6*, *hta8 hta9 hta11*, and *pie1* roots: (A–**F**) ChIP-qPCR analysis of H3K27me3 enrichment in *MSRB6*, *CYP735A2*, *APX4*, *UQCRH*, *HY2*, and *FRO2* genes in WT, *arp6*, *hta8 hta9 hta11*, and *pie1* roots. Primer set numbers are the same as those listed in Figure 5. Error bars indicate ± SD (*n* = 3 replicates). Asterisks above the columns indicate statistically significant differences (* *p* < 0.05, ** *p* < 0.01) determined by Student’s *t* test.

We also detected H3K27me3 enrichment in oxidoreductase activity-related genes in WT, *arp6*, *hta8 hta9 hta11*, and *pie1*. By contrast, H3K27me3 enrichment in this region of oxidoreductase activity-related genes was significantly increased in *hta8 hta9 hta11*, and *pie1* compared with WT and *arp6* (Figure 8A–F). Compared to WT, these oxidoreductase activity-related genes’ reduced expression in *arp6*, *hta8 hta9 hta11*, and *pie1* correlated with decreased H3K4me3 enrichment and increased H3K27me3 enrichment.

## 3. Discussion

Epigenetic mechanisms of genome regulation include DNA methylation modification, histone modification, and chromatin remodeling [27,34,56,57]. The nucleosome forms the structure and basic unit of eukaryotic chromatin [29,30]. The chromatin remodeling complex regulates gene transcription and expression by changing the nucleosome composition, packaging, and positioning [34]. At present, several studies have reported that the SWR1 chromatin remodeling complex plays an important role in regulating gene expression by incorporating the histone variant H2A.Z into nucleosomes. For example, SWR1 interacts with the ERECTA-mediated signaling pathway to promote *Arabidopsis* resistance to *Sclerotinia sclerotiorum* (*S. sclerotiorum*) by affecting *WRKY33* expression and its target *YDA DOWNSTREAM* (*YDD*) genes [32]. SWR1 also coordinates with the ERECTA signaling pathway to control *Arabidopsis* ovule development and inflorescence architecture [42]. In addition, several studies have indicated that H2A.Z and histone modification regulate gene expression in a genome-wide manner [39,55]. However, less is known about the function of these epigenetic regulations in root growth and development. In this study, the H2A.Z deposition-deficient mutants, *arp6*, *pie1*, and *hta8 hta9 hta11*, exhibited a lower primary root length and more LRs than those in WT (Figure 1A–C). Our results are similar to a previous study [58]. SWR1 reportedly affects inflorescence architecture by promoting cell proliferation in the pedicel cortex and cell elongation in the pedicel epidermis [42]. We found that the inhibition of *arp6*, *pie1*, and *hta8 hta9 hta11* root lengths may be caused by reduced cell numbers and shorter cell lengths in the meristematic and maturation zones (Figure 1D–G), respectively. Therefore, the key SWR1 complex component and H2A.Z variant play important roles in root growth and development.

In plants, reactive oxygen species are byproducts of various metabolic reactions, such as electron transfer chains or redox reactions in chloroplasts or mitochondria [59]. Increasing evidence has demonstrated that redox systems play an important role in regulating cell signal transmission, light morphogenesis, and plant growth and development [1,13]. The RNA-seq results revealed that down-regulated genes were enriched in oxidoreduction-driven active transmembrane transporter activity (Figure 2D). Since ROS maintain the balance between cell proliferation and elongation [7], we performed DAB and NBT staining to examine ROS accumulation in WT and H2A.Z deposition-deficient mutants. Our results showed that *arp6*, *pie1*, and *hta8 hta9 hta11* seedlings accumulated more H_2_O_2_ and O_2_^−^ in their roots than WT (Figure 4). Our results showed that the chromatin complex SWR1 affects the accumulation of ROS in roots, and ROS may be one of the factors affecting root elongation. 

**Figure 9:**
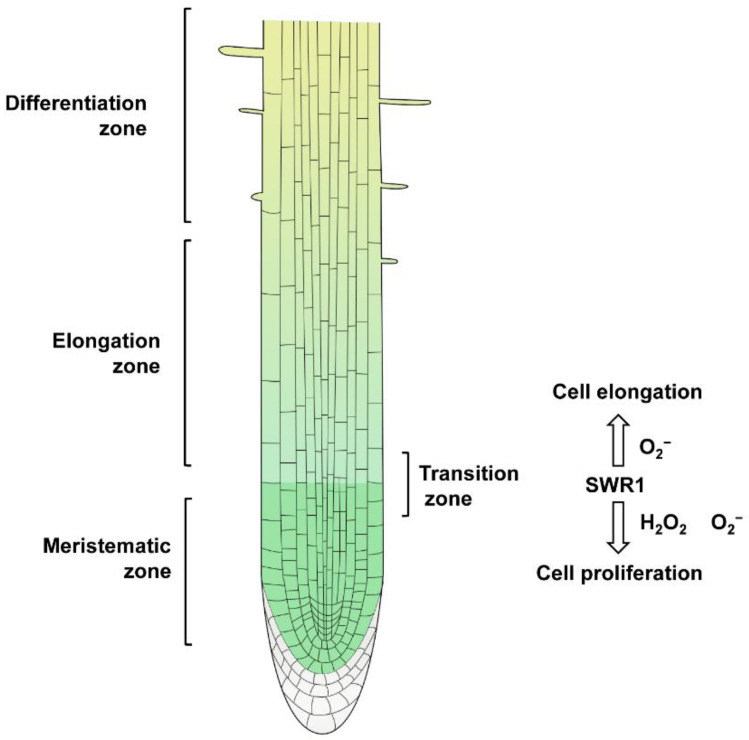
Chromatin remodeling complex SWR1 regulates root growth and development by affecting ROS accumulation.

*MSRB6* [46], *CYP735A2* [47,48], *APX4* [49], *UQCRH* [50], *HY2*, and *FRO2* are involved in oxidation–reduction processes [51,52,53]. Epigenetic mechanisms such as histone modification and DNA methylation reportedly affect gene transcription and expression [12,33,55]. For example, SWR1 promotes the H2A.Z deposition and H3K4me3 modification of *YDD* genes, facilitating plant immunity in response to *S. sclerotiorum* [32]. By contrast, H2A.Z and H3K4me3 play antagonistic roles in regulating anthocyanin biosynthesis under drought and high-light stresses [33]. The role of H2A.Z in downstream auxin signaling transduction has been investigated. H2A.Z can affect auxin-related phenotypes such as LR formation and gravitropism [58]. SDG2-mediated H3K4me3 methylation plays a distinctive role in regulating root meristem activity [12]. However, less is known about the function of these epigenetic relation types in root growth and development. We found reduced H2A.Z enrichment in oxidoreductase activity-related genes, indicating that the deposition of H2A.Z on oxidoreductase activity-related genes depends on key SWR1 and H2A.Z histone variant components (Figure 5). Furthermore, the reduced deposition of Pol II and H3K4me3 (Figure 6 and Figure 7) and the elevated level of H3K27me3 in oxidoreductase activity-related genes in H2A.Z deposition-deficient mutants *arp6*, *pie1*, and *hta8 hta9 hta11* (Figure 8) contributed to the expression level changes in these mutants’ oxidoreductase activity-related genes. However, the deposition of Pol II and H3K27me3 at MSRB6 did not change significantly, possibly because the repression of this gene is post-transcriptional or post-translational. Moreover, H2A.Z, Pol II, H3K4me3, and H3K27Me3 cannot be deposited at position 1, which corresponds to the promoter region of *APX4* and *HY2*. Fewer H2A.Z, Pol II, H3K4me3, and H3K27Me3 of *APX4* and *HY2* in this region may cause this trend. In summary, our results show that the chromatin remodeling complex SWR1 affects the expression of oxidoreductase activity-related genes and leads to ROS accumulation in roots, thereby inhibiting their growth and development (Figure 9). 

## 4. Materials and Methods

### 4.1. Plant Materials and Growth Conditions

Seeds of *Arabidopsis* (*Arabidopsis thaliana*) wild-type (Col-0 ecotype) and mutants *arp6* (Garlic_599_G03), *pie1* (*pie1-5*, *SALK_096434*), *hta8 hta9 hta11* (provided by Yafei Wang at Northwest A&F University, Yangling 712199, China) [60]. Seeds were planted in 1/2 Murashige and Skoog (MS) medium and grown under a 16 h light/8 h dark photoperiod at 22 °C.

### 4.2. RNA-seq and Analysis of Differentially Expressed Genes

We isolated the total RNA from 10-day-old WT, *arp6*, *pie1*, and *hta8 hta9 hta11* roots using a plant RNA extraction kit (OMEGA, Shanghai, China) following the manufacturer’s protocol. Sequencing and data processing were conducted as previously described [61]. We used the TAIR10 *Arabidopsis thaliana* as the reference genome and STAR v2.5.0 to align the clean reads with it. We processed the alignment results using the SourceForge Subread package feature Count v1.5.0 for gene quantification. Finally, edgeR v3.12.0 was used to identify the differentially expressed genes (fold change ≥ 2; a value of FDR ≤ 0.05 was considered statistically significant) between samples. We performed GO and KEGG analysis of differential genes using TBtools v1.09 software [62].

### 4.3. Quantitative Real-Time PCR Analysis 

RNA was reversed-transcribed using AMV reverse transcriptase (Takara, Japan) according to the manufacturer’s instructions. We performed quantitative real-time PCR was performed based on the SYBR Premix Ex Taq II system (Takara, Japan) and Bio-Rad Real-Time PCR system. The reaction volume was 20 µL and contained 10 µL of 2× SYBR Premix, 8.2 µL of RNase-free water, 1 µL of template, and 0.4 µL of each specific primer (Table S3). The reaction was performed under the following parameters: 95 °C for 30 s; 40 cycles of 95 °C for 5 s and 60 °C for 34 s; 95 °C for 15 s. *HK2* (*AT4G26410*) was used as a reference gene. We performed three biological replicates, and three technical replicates confirmed every biological replicate. 

### 4.4. ChIP-qPCR

We used 1 g of 10-day-old *Arabidopsis* root for each ChIP experiment. We fixed the *Arabidopsis* root sample in fix buffer (0.4 M sucrose, 10 mM Tris-HCl (pH 8.0), 1 mM Phenylmethylsulfonyl fluoride (PMSF), 1 mM EDTA, 1% formaldehyde (*w*/*v*)) for 10 min using vacuum infiltration and stopped in 2 M glycine. Cross-linked chromatin was fragmented with 0.2 units of micrococcal nuclease (Sigama) in 1 mL of MNase digestion buffer (10 mM Tris-HCl (pH = 8.0), 50 mM NaCl, 1 mM mercaptoethanol, 0.1% Nonidet P-40, 1 mM CaCl_2_, and 1×protease inhibitor cocktail (Roche)) for 10 min. Digestion was stopped using 5 mM EDTA (pH = 8.0). ChIP was performed using H2A.Z polyclonal antibodies (provided by R. Meagher at the University of Georgia, Athens, GA), polyclonal anti-H3K4me3 (07-473; Millipore), Pol II antibodies (Abcam, ab817), and anti-H3K27me3 (Millipore, 07-449). All ChIP experiments were performed in a buffer containing 1% Triton X-100, 1× protease inhibitor mixture, 10 mM Tris-HCl (pH = 8.0), 5 mM EDTA (pH = 8.0), and 150 mM NaCl. We analyzed the relative enrichment of associated DNA fragments by quantitative PCR (qPCR). The 100–300 bp sequence upstream of the TSS was designed as primer-1. The 100-200 bp sequence around the TSS region was designed as primer-2, and the 100-300 bp sequence downstream of the TSS was designed as primer-3 in the ChIP-qPCR experiment. Table S3 lists the oligonucleotide sequences used for target DNA detection and quantification in ChIP experiments. The ChIP experiment results were calculated by the fold enrichment method, as described in previous studies [33].

### 4.5. Histochemical Assays

To detect ROS in the sample roots, the seedlings were steeped in 0.5 mg/mL of 3,3-diaminobenzidine (DAB, aladdin) in 50 mM of Tris-HCl (pH = 5.0) for 12 h in darkness, or 0.5 mg/mL nitroblue tetrazolium (NBT, Sigma) in 20 mM of K phosphate/0.1 M NaCl at pH 6.1 for 15 min. We gently transferred the seedlings to 75% ethanol and boiled them for 15 min. We used Image J software to measure the grayscale of DAB and NBT staining in the WT and mutants. Average DAB or NBT intensities from WT roots were set to 100%. Other roots’ DAB or NBT intensities were calculated as the WT percentage.

### 4.6. Statistical Analysis

We conducted all experiments with three biological repeats and three technical repeats. The data were shown as means ± standard errors (SD; *n* = 3). Student’s *t* test was used to analyze significant differences between WT and each mutant by GraphPad Prism 8.0.1 software. Asterisks indicate significant differences * *p* < 0.05, ** *p* < 0.01.

## 5. Conclusions

In this study, we found that H2A.Z-deficient *Arabidopsis* mutant plants exhibit shorter root lengths and fewer LRs compared with those in wild-type plants. Our results showed that ROS accumulation was increased in the roots of H2A.Z deposition-deficient mutants. Furthermore, oxidoreductase activity-related genes’ reduced expression level in H2A.Z deposition-deficient mutants is associated with decreased H2A.Z deposition, H3K4me3 histone modification, RNA Pol II enrichment, and increased H3K27me3 histone modification. This study enhanced our understanding of the chromatin remodeling complex SWR1 function in root growth and development and highlighted the role of chromatin in gene regulation. 

## Data Availability

All data analyzed during this study are included in this article and its additional files.

## Supplementary Materials

The following supporting information can be downloaded at https://www.mdpi.com/article/10.3390/plants12040940/s1. Figure S1. Expression patterns of the protein kinases and transcription factors among the differentially expressed genes (DEGs): (A,B) The protein kinases (A) and transcription factors (B) among the up-regulated genes. (C,D) The protein kinases (C) and transcription factors (D) among the down-regulated genes. Table S1. Statistics of sequence mapped with the reference genome. Table S2. DEGs of WT and H2A.Z deposition-deficient mutants. Table S3. Primers used in this study.
