# Supplementary figures and images for: Chromatin Remodeling Complex SWR1 Regulates Root Development by Affecting the Accumulation of Reactive Oxygen Species (ROS)
Source: Plants (Basel). 2023 Feb 19;12(4):940. doi: 10.3390/plants12040940 (PMC9964059; doi:10.3390/plants12040940)

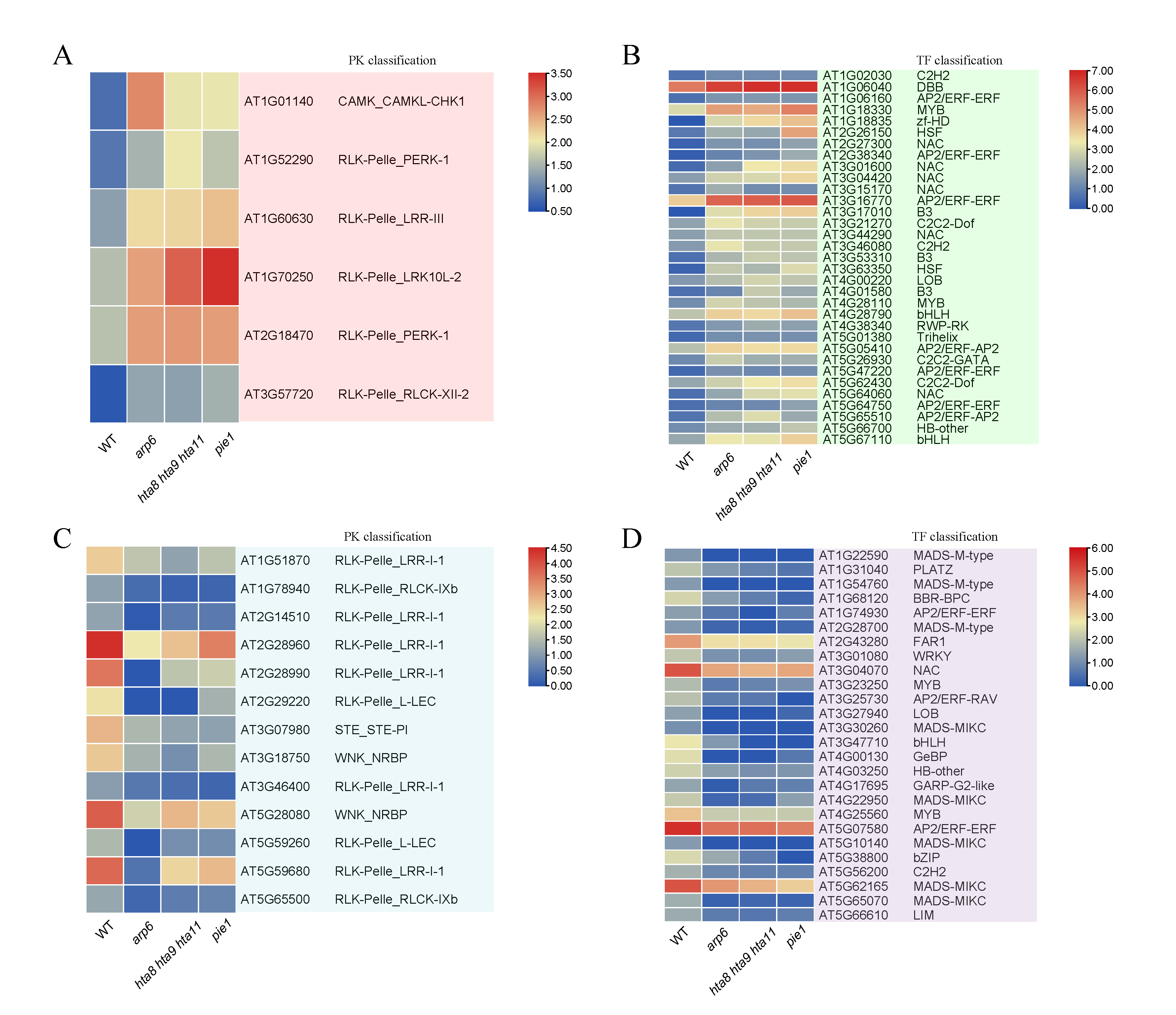

Supplement: Supplementary file 1 [file plants-12-00940-s001.zip › Supplementary Figure S1.tiff]
